# The combined effect of two mutations that alter serially homologous color pattern elements on the fore and hindwings of a butterfly

**DOI:** 10.1186/1471-2156-8-22

**Published:** 2007-05-11

**Authors:** Antónia Monteiro, Bin Chen, Lauren C Scott, Lindsey Vedder, H Joop Prijs, Alan Belicha-Villanueva, Paul M Brakefield

**Affiliations:** 1 Department of Biological Sciences, University at Buffalo, 109 Cooke Hall, Buffalo, NY 14228, USA; 2 Section of Evolutionary Biology, Institute of Biology, Leiden University, P.O. Box 9516, 2300 RA Leiden, The Netherlands; 3 Department of Ecology and Evolutionary Biology, Yale University, P.O. Box 208106, New Haven, CT 06520-8106, USA

## Abstract

**Background:**

The ability for serially homologous structures to acquire a separate identity has been primarily investigated for structures dependent on Hox gene input but is still incompletely understood in other systems. The fore and hindwings of butterflies are serially homologous structures as are the serially homologous eyespots that can decorate each of these wings. Eyespots can vary in number between fore and hindwings of the same individual and mutations of large effect can control the total number of eyespots that each of the wings displays. Here we investigate the genetics of a new spontaneous color pattern mutation, *Missing*, that alters eyespot number in the nymphalid butterfly, *Bicyclus anynana*. We further test the interaction of *Missing *with a previously described mutation, *Spotty*, describe the developmental stage affected by *Missing*, and test whether *Missing *is a mutant variant of the gene *Distal-less *via a linkage association study.

**Results:**

*Missing *removes or greatly reduces the size of two of the hindwing eyespots from the row of seven eyespots, with no detectable effect on the rest of the wing pattern. Offspring carrying a single *Missing *allele display intermediate sized eyespots at these positions. *Spotty *has the opposite effect of *Missing*, i.e., it introduces two extra eyespots in homologous wing positions to those affected by *Missing*, but on the forewing. When *Missing *is combined with *Spotty *the size of the two forewing eyespots decreases but the size of the hindwing spots stays the same, suggesting that these two mutations have a combined effect on the forewing such that *Missing *reduces eyespot size when in the presence of a *Spotty *mutant allele, but that *Spotty *has no effect on the hindwing. *Missing *prevents the complete differentiation of two of the eyespot foci on the hindwing. We found no evidence for any linkage between the *Distal-less *and *Missing *genes.

**Conclusion:**

The spontaneous mutation *Missing *controls the differentiation of the signaling centers of a subset of the serial homologous eyespots present on both the fore and the hindwing in a dose-dependent fashion. The effect of *Missing *on the forewing, however, is only observed when the mutation *Spotty *introduces additional eyespots on this wing. *Spotty*, on the other hand, controls the differentiation of eyespot centers only on the forewing. *Spotty*, unlike *Missing*, may be under Ubx gene regulation, since it affects a subset of eyespots on only one of the serially homologous wings.

## Background

Modularity of body plans, and of serially repeated structures is widespread in the animal kingdom [1]. Examples of modular structures include vertebrae [2], teeth [3], limbs [4], digits [5], arthropod body segments [6], *C. elegans *terminal rays [7], insect fore and hindwings [8-10], and butterfly eyespot patterns [11-13]. One of the key questions driving research in the field of modularity is to understand how such modules acquire the ability to differentiate into more or less distinct structures [14-16]. This differentiation and specialization of repeated body parts has, in the case of arthropod's body segments, increased through evolutionary time [17] presumably facilitating the radiation of these organisms into different environments.

The two pairs of wings on a butterfly are serially repeated structures, as are several of the pattern elements present within each wing [18]. Pattern elements such as eyespots, for instance, can occur in each of the wing subdivisions, the **wing cells**, delineated by veins. Nijhout [18] proposed that the diversity of butterfly color patterns seen today results from the presence or absence, or through the modification of the size, shape, color or position of these serially homologous pattern elements within each wing cell. While the Hox gene, Ultrabithorax (Ubx), appears to be responsible for allowing the hindwing to acquire different wing patterns from the forewing [8], the genes and mechanisms by which particular serially homologous elements within each wing can acquire a separate identity from the other elements, however, are still unknown. Here we analyze the phenotypic effects of two spontaneous mutations, *Missing *and *Spotty *that affect the development of two serially homologous eyespots independently of the remaining eyespots on the wings.

The nymphalid butterfly *Bicyclus anynana *normally displays two marginal eyespots on both the dorsal and ventral sides of the forewing and seven eyespots on the ventral hindwing. Spontaneous [19,20] and X-ray induced mutations [12] can alter the number of eyespots that appear on the wings surfaces, producing substantial departures from the wild type pattern. The spontaneous mutation *Spotty *was previously shown to be a single segregating factor of codominant effect [21]. Spotty homozygotes have two extra eyespots on both the ventral and dorsal surfaces on the forewings of *B. anynana*. These extra eyespots occur in wing cells between those carrying the wildtype anterior and posterior eyespots. Spotty heterozygotes have either reduced sizes for both extra eyespots, or have one of the eyespots missing and the other eyespot present but reduced in size. This mutation, despite only having been analyzed in a qualitative fashion, appears to show a very localized effect in controlling the eyespot developmental program in a subset of the wing cells. *Missing *is a new spontaneous mutation that has a very similar phenotype to another x-ray induced mutation, *3+4*, previously described [12]. Contrary to *Spotty*, this mutation **removes **two eyespots from the **hindwing**, but the wing cells affected by *Missing *are homologous to those affected by *Spotty *on the forewing.

Here we investigate the phenotypic effects of *Missing *when crossed with either *Missing*, wildtype, or *Spotty *in order to describe the mutation in homozygote and in heterozygote condition and also to investigate whether *Missing *and *Spotty*, which when in isolation only seem to affect the hindwing or forewing pattern, respectively, show any interaction when placed together in the same individual. We additionally test whether during the imaginal disc stage the Distal-less and Engrailed transcription factors are absent from the centers of the hindwing eyespots that don't develop in adults of the Missing stock. We select one of these genes, *Distal-less*, as a possible target of the *Missing *mutation, and test whether this candidate gene is associated to the Missing phenotype by means of a linkage association study.

## Results

### Extreme missing individuals have low fitness

**Figure 1:**
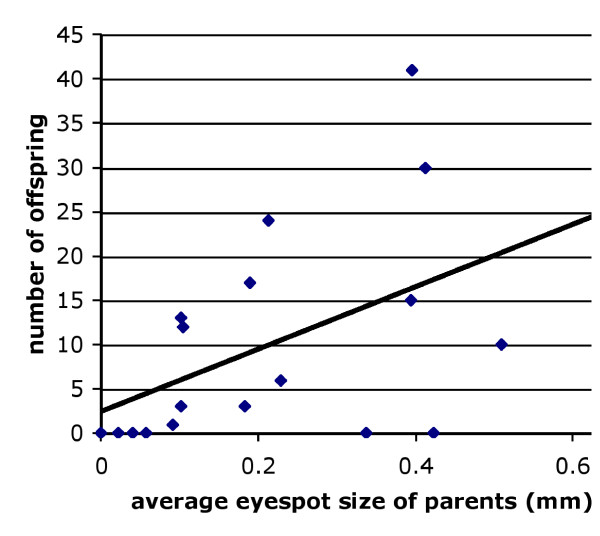
**Extreme Missing individuals have fewer offspring**. Relationship between the average size of eyespots 3 and 4 in parents carrying absent or very small eyespots at these positions, and the number of offspring produced. The proportion of variation explained by the regression line (R^2^) is 0.24 and its slope is significantly different form zero (P = 0.028).

Missing individuals show considerable variation in their phenotype, even after intensive selection for a pure homozygote stock. This variation is represented by the presence of very small dots marking the center of the hindwing eyespots, in wing cells 3 and 4, in a large proportion of the individuals. For our crosses we selected mostly extreme looking individuals, but sometimes included individuals that displayed a differentiated but tiny eyespot pupil, and sometimes a very narrow gold ring. When no black scales were visible, but a pupil was clearly differentiated, the diameter of the eyespot was given as the diameter of the white pupil. We observed that families whose parents had an extreme "Missing" phenotype produced few offspring, compared to families where the average size of eyespots 3 and 4 in the parents was less extreme (Figure 1). After surveying the distribution of eyespot sizes in the offspring we decided that some of the putative "Missing × Missing" crosses were probably crosses between *Missing/Missing *and *Missing*/*Wildtype *heterozygotes and excluded them from future comparisons. In practice, this meant that we applied a threshold of 0.3 mm to the maximal size that each of the two hindwing eyespots can display in order for that individual to be called a Missing phenotype. It is also likely that other modifier loci are contributing to additional variation in the size of the eyespots targeted by Missing and possibly also contributing to variation in fitness.

### The effect of *Missing *on the hindwing

**Figure 2:**
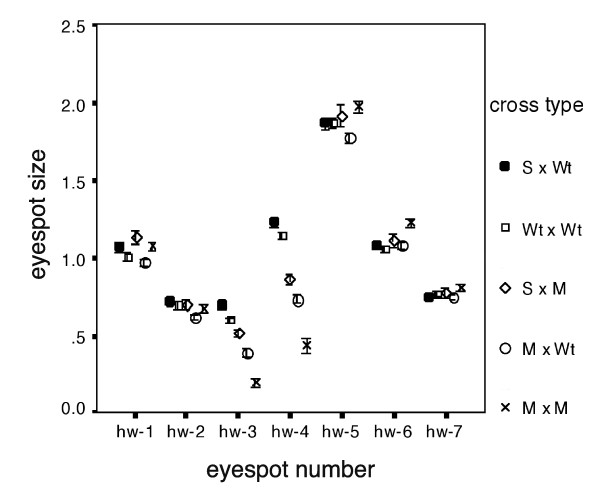
**The effect of *Missing *is restricted to two of the hindwing eyespots**. Average eyespot size for each of the seven eyespots on the hindwing for five different set of crosses (sexes and families combined). Spotty × Wildtype (filled circle), Wildtype × Wildtype (square), Spotty × Missing (bullseye), Missing × Wildtype (empty circle), and Missing × Missing (cross). Error bars represent 95% confidence intervals for the mean.

Crosses of Missing with Missing (Figure 2) produced offspring that displayed some variation in their hindwing phenotype, but overall displayed reduced or absent eyespots at positions 3 and 4. Hybrids resulting from crosses of Missing with Wildtype produced intermediate sized eyespots 3 and 4 relative to those from Wildtype*Wildtype and Missing × Missing eyespots (Figure 2). Analysis of variance for the size of each of the hindwing eyespots among the three types of cross mentioned above showed that the effect of *Missing *is localized to positions 3 and 4 on the hindwing (Table 1). Sex and family are also factors that explain a significant proportion of the variance in the data (Table 1). This mutation has a codominant effect relative to Wildtype as well as a localized effect on the hindwing.

**Table 1:** The effect of *Missing *on the hindwing.

	**n**	**Cross type (df = 2)**	**p**	**Sex (df = 1)**	**p**	**Family (df = 19)**	**p**
**hw eye 1**	731	1.512	0.246	23.339	0.000	15.194	0.000
**hw eye 2**	730	1.960	0.168	18.430	0.000	14.342	0.000
**hw eye 3**	731	25.766	0.000	6.749	0.010	15.455	0.000
**hw eye 4**	731	47.945	0.000	47.604	0.000	12.604	0.000
**hw eye 5**	730	2.306	0.127	178.781	0.000	18.563	0.000
**hw eye 6**	727	3.151	0.066	289.971	0.000	18.311	0.000
**hw eye 7**	721	0.722	0.499	170.101	0.000	16.700	0.000

F-values and probabilities from an analysis of variance to explore whether there is a difference in hindwing eyespot size among three types of cross: Wild type × Wild type, Wild type × Missing and Missing × Missing. Sex and Family (random factor nested within cross-type) were used as additional factors. Only main effects for factors were calculated.

**Figure 3:**
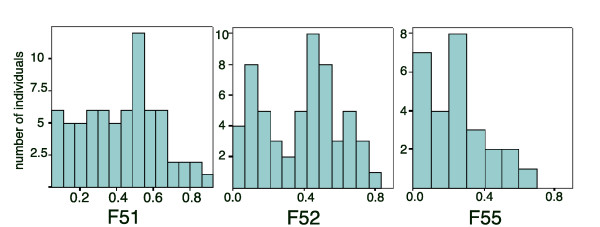
**Backcross distribution plots**. The distribution of average eyespot sizes for eyespots 3 and 4 for offspring from the three back-crosses of Wiltype/Missing × Missing (Families F51, F52, and F55). Error bars represent 95% CI for the means.

When *Missing/Wildtype *individuals were backcrossed to Missing there appeared to be a bi-modal distribution of phenotypes, especially pronounced in one of the largest families (Figure 3, F52). There appears to be, however, fewer offspring under the first peak of the bi-modal distributions. This may relate to the fact that several extreme *Missing/Missing *genotypes may have not survived to adulthood. In all, these results suggest that the *Missing *mutation(s) is likely segregating at a single major genetic locus.

### The effect of *Spotty *on the forewing

The effect of the *Spotty *mutation on the forewing was previously described by Brakefield and French [21], who concluded that this mutant had a codominant effect relative to Wildtype. Spotty homozygotes display two well-developed eyespots in positions 3 and 4 on the forewing, and heterozygotes display two intermediate sized eyespots at these positions, or sometimes a single smaller eyespot at either position 3 or position 4.

### The effect of *Missing *on the forewing

**Figure 4:**
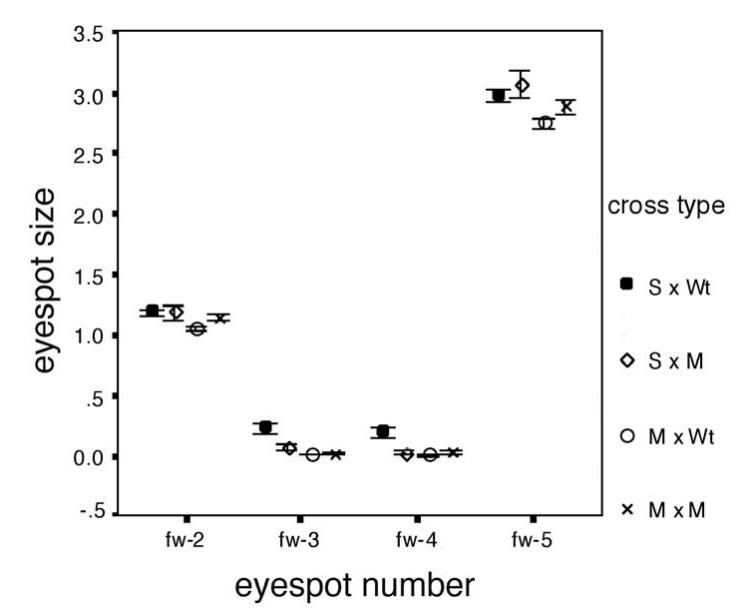
***Missing *also reduces the size of forewing eyespots**. Average eyespot size for each of the four eyespots on the forewing for four different set of crosses (sexes and families combined): Spotty × Wildtype (filled circle), Spotty × Missing (bullseye), Missing × Wildtype (empty circle), and Missing × Missing (cross). Error bars represent 95% confidence intervals for the mean.

Wildtype individuals normally do not display eyespots in positions 3 and 4 on the forewing (not shown). Missing*Missing and Missing*Wildtype crosses also did not display eyespots at these positions (Fig. 4).

### The effect of *Spotty *on the hindwing

The size of the hindwing eyespots between offspring of Spotty × Wildtype versus offspring from Wildtype*Wildtype crosses (Fig. 2) are not significantly different for any of the eyespots (Table 2), indicating that *Spotty *only affects the number and size of eyespots located on the forewing.

**Table 2:** The effect of *Spotty *on the hindwing.

	**n**	**Cross type (df = 1)**	**P**	**Sex (df = 1)**	**p**	**Family (df = 8)**	**p**
**hw eye 1**	505	0.633	0.449	9.478	0.002	25.289	0.000
**hw eye 2**	505	0.053	0.823	5.785	0.017	35.354	0.000
**hw eye 3**	505	4.056	0.078	19.695	0.000	19.433	0.000
**hw eye 4**	505	1.028	0.340	51.158	0.000	16.781	0.000
**hw eye 5**	505	0.156	0.704	135.204	0.000	43.546	0.000
**hw eye 6**	505	0.001	0.978	206.472	0.000	38.923	0.000
**hw eye 7**	505	0.274	0.615	117.239	0.000	38.247	0.000

F-values and probabilities from an analysis of variance to explore whether there is a difference in hindwing eyespot size between two types of cross: Spotty × Wild type, and Wild type × Wildtype. See additional info in legend of Table 1.

### The combined effects of *Spotty *and *Missing *on the forewing and on the hindwing

Crosses of homozygous *Missing *with homozygous *Spotty *showed that these mutations interact and display an additive effect on the forewing but not on the hindwing. When *Missing *is combined with *Spotty*, the previously undetected effect of *Missing *on the forewing (see above) becomes visible in the *Spotty *heterozygote background. Forewing eyespots 3 and 4 are smaller in Missing × Spotty offspring relative to Wildtype × Spotty offspring (Figure 4; Table 3). There was no difference in the size of the flanking eyespots (eyespots 2 and 5) in these two types of cross (Table 3). These results indicate that *Missing *has the effect of reducing the size of eyespots 3 and 4 on both hindwings and forewings.

**Table 3:** The effect of *Missing *on the forewing in a Spotty background.

	**n**	**Cross type (df = 1)**	**P**	**Sex (df = 1)**	**p**	**Family (df = 6)**	**p**
**fw eye 2**	259	0.042	0.844	19.956	0.000	27.303	0.000
**fw eye 3**	259	5.773	0.042	26.794	0.000	3.272	0.004
**fw eye 4**	260	10.664	0.009	38.542	0.000	2.172	0.046
**fw eye 5**	261	0.218	0.656	505.787	0.000	23.066	0.000

F-values and probabilities from an analysis of variance to explore whether there is a difference in forewing eyespot sizes between two types of cross: Spotty × Wild type, and Spotty × Missing. See additional info in legend of Table 1.

When the effect of *Spotty *was analyzed on the hindwing, by comparing eyespot sizes from Spotty × Missing versus Wildtype × Missing offspring (Figure 2), there was no significant effect of cross-type detected for any of the eyespots (Table 4). To be able to have more power in detecting the effect of *Spotty *on the hindwing, we also investigated whether the effect of the *Spotty *allele, in Spotty × Wildtype and Spotty × Missing offspring combined, resulted in larger hindwing eyespot sizes relative to the Wildtype allele in Wildtype × Wildtype, or Wildtype × Missing offspring combined. Despite the larger sample size (n = 803), there was still no detectable significant effect of *Spotty *in increasing hindwing eyespot size (data not shown).

**Table 4:** The effect of *Spotty *on the hindwing in a Missing background.

	**n**	**Cross type (df = 1)**	**p**	**Sex (df = 1)**	**p**	**Family (df = 8)**	**p**
**hw eye 1**	298	3.834	0.095	23.124	0.000	26.999	0.000
**hw eye 2**	298	2.360	0.171	16.580	0.000	16.060	0.000
**hw eye 3**	298	1.804	0.225	5.395	0.021	22.767	0.000
**hw eye 4**	298	1.749	0.230	55.719	0.000	14.663	0.000
**hw eye 5**	298	1.623	0.246	121.377	0.000	16.063	0.000
**hw eye 6**	298	0.160	0.701	81.218	0.000	10.900	0.000
**hw eye 7**	297	0.350	0.574	41.664	0.000	16.560	0.000

F-values and probabilities from an analysis of variance to explore whether there is a difference in hindwing eyespot size between two types of cross: Missing × Wild type, and Missing × Spotty. See additional info in legend of Table 1.

### Immunohistochemistry

**Figure 5:**
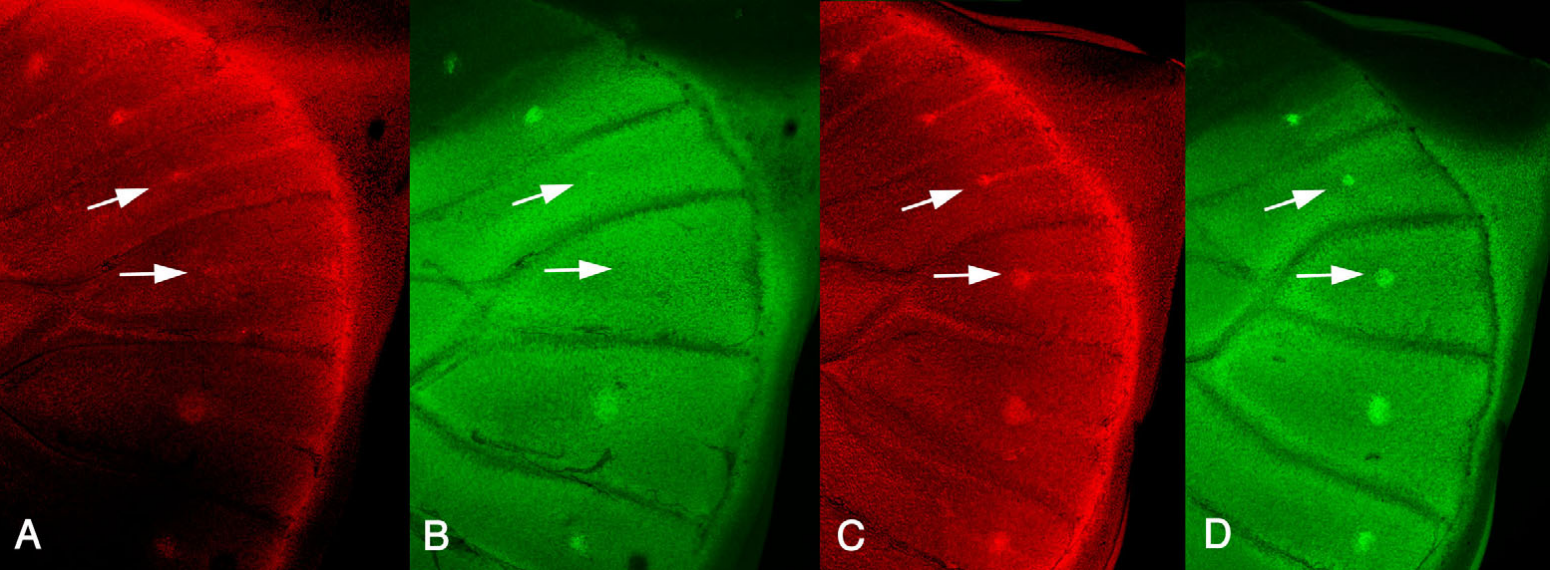
**Distal-less and Engrailed are expressed at low levels in two of the hindwing eyespot centers in Missing**. Distal-less (red) and Engrailed (green) immunolocalizations in late fifth instar larval wing discs of a Missing and a Wildtype individual. A, B) Missing individual showing very low levels of Dll and En in the eyespot foci of wing cells 3 and 4 (arrows); C, D) Wildtype individual showing normal levels of Dll and En protein levels in the foci.

Late larval hindwing discs of individuals taken from the Missing stock displayed a range of *Distal-less *and *engrailed *expression patterns in the 3^rd ^and 4^th ^wing cells, from the absence of both these proteins in the future eyespot centers (Figure 5), to the presence of small differentiated foci, expressing both proteins (not shown). The expression of *Distal-less *along the intervenous stripes was present in all hindwing cells.

### Association study

We identified an informative SNP (C/A) in a recognition sequence for the *Bse*NI restriction enzyme (NCCAGT) in the Dll 5'UTR fragment analyzed for the four grandparents. The wildtype grandparent was homozygous CCCAGT and the *Bse*NI enzyme cut the amplified PCR fragment into two fragments, 451 bp and 239 bp long, respectively. The Missing grandparent was homozygous CCAAGT and thus was not cut by the *Bse*NI enzyme. The F1 male parent was an expected heterozygote (CCCAGT/CCAAGT) and had the expected heterozygote restriction pattern; whereas the Missing female that was crossed with the F1 male had a similar genotype as the Missing grandmother, and was not cut by the restriction enzyme.

We tested two hypotheses: 1) If the *Dll *polymorphism is linked to the *Missing *mutation then we expect to find the majority of backcross individuals with the smallest or with absent eyespots to be homozygote for the CCAAGT genotype and display the "no-cut" pattern; and the majority of the individuals with the largest eyespots to display the "heterozygote-cut" restriction pattern on the gel. 2) On the other hand if the *Dll *polymorphism is not linked to the *Missing *mutation then we expect approximately 50% of backcross individuals with the smallest eyespots to have the "no-cut" pattern, and 50% to have the "heterozygote-cut" pattern. The same frequencies will be expected for the individuals with the largest eyespots.

From the 15 individuals that were genotyped with the smallest eyespots (0.06 mm average diameter ± 0.02 mm SE) we obtained 7 individuals (2 females and 5 males) that were not cut by the enzyme, and 8 individuals (4 females and 4 males) that were cut by the enzyme. From the 15 individuals that were genotyped with the largest eyespots (0.51 mm average diameter ± 0.03 mm SE), 10 individuals (2 females and 8 males) were not cut by the enzyme, and 5 individuals (3 females and 2 males) were cut by the enzyme. These results clearly support the hypothesis of no linkage between *Dll *and *Missing *genes.

## Discussion

The series of crosses clearly show that *Missing*, is a co-dominant mutation that has the dramatic effect of removing or reducing the size of two eyespots on the hindwing when in homozygote and heterozygote condition, respectively. *Missing *has the additional effect of reducing the size of the eyespots present in a homologous position on the forewing. The subtle effect of the *Missing *mutation on the forewing is not easily observed by eye and was only borne out because of the detailed quantitative approach used here. *Spotty*, on the other hand, is a mutation that introduces two eyespots on the forewing, and has no effect on the size of the homologous eyespots on the hindwing.

Selector genes such as Ubx, that are expressed only on the hindwings of insects, including butterflies, have been shown to affect the size of eyespots on the hindwing relative to their homologous counterparts on the forewing [8][22]. Ubx-dependent eyespot size changes in the butterfly *Junonia (Precis) coenia *involve quantitative changes both in the signaling component as well as in the response components of the eyespot differentiation mechanism [8]. From our analysis it appears that the qualitative effect of the *Spotty *allele on eyespot number is also regulated in a Hox gene-dependent fashion. *Spotty *is selectively expressed in the forewing, but not in the hindwing, indicating that a putative hindwing repressor (such as Ubx) modulates the effect of this allele. On the other hand, the *Missing *allele appears to exert its action on the eyespot developmental program in a Ubx gene-independent fashion. *Missing *will affect the size of homologous eyespots in both forewing and hindwings simultaneously.

It still remains to be determined whether these two mutations are genetically linked, perhaps representing alleles of the same gene. Crosses performed between Missing and Spotty individuals, followed by backcrosses to Missing, Wildype, or Spotty, were performed in order to try and analyze the shape of the backcross distributions. The shape of these distributions would indicate the likely linkage status of the *Missing *and *Spotty *genes. If the distributions had a clear two-peaked shape, this would indicate likely linkage, and the presence of two main genotypic classes (*M/S *and *M/M *for backcrosses to Missing for instance). If, on the other hand, the distributions were more normally distributed, i.e, reflecting the sum of four normal distributions, each representing a genotypic class, centered around different mean values (*M/M*, +/+; *M/M*, *S*/+; +/*M*, +/+; and +/*M*, *S*/+), then this would indicate that *Missing *and *Spotty *were found either on different linkage groups, or in the same linkage group but far apart. Unfortunately, there were two major complications to the successful completion of these analyses. First, the backcross distribution of Missing/Spotty hybrids crossed to Missing produced a skewed distribution against the zero eyespot diameter along the x-axis, where a series of Missing-like individuals were clustered. These skewed distributions cannot be readily analyzed with standard mixture models. Second, there was a strong family effect in that while some families produced a strong two-peaked distribution, other families didn't.

The immunohistochemistry showed that the *Missing *mutation, similarly to the *3+4 *mutation previously described [12], affects the complete differentiation of the group of signaling cells at the center of the future eyespots. The focal cells, marked by the domains of *Dll *and *en *expression, are either a small cluster or do not differentiate at all, and as a result, there is no signaling during the pupal stage of eyespot differentiation, no visible white pupil, and no color rings of scales on the adult wing.

*Distal-less *is known to be one of the first genes to be expressed in the eyespot pupils during the fifth instar larval wing disc stage [23-25]. In *Drosophila*, this gene is also the first gene to be expressed in the future position of the ventral appendages of the fly. It acts as a selector gene that works in a fairly context-independent fashion since its ectopic activation in the leg disk of *Drosophila *results in a complete leg duplication [26]. Because of these similarities we decided to test whether a putative mutation in the regulatory region of this gene may be causing the Missing phenotype. The association study concluded that the *Missing *mutation is not linked to *Distal-less*. Alternative genes to test would include *Notch*, also shown to be expressed in the larval wing disc, in the eyespot foci, and recently shown to be expressed slightly earlier in development in these cells relative to *Distal-less *[23], or other genes involved in the focus differentiation pathway, but putatively already acting downstream of *Dll *and *Notch *(reviewed in [20,27]). Given that we are looking for a single gene mutation, a more sensible approach to identify this gene in the future may be to take a positional mapping study, with markers positioned all along the genome, until a candidate genomic regions is found to be associated with the mutation. Progress in the development of genomic tools for *Bicyclus *may allow this to be done in the near future [28].

## Conclusion

We described the phenotypic effects of two mutations that affect the development of two of the serial homologous eyespots present in both fore and hindwings of *B. anynana*. Both mutations have a co-dominant effect relative to their wild-type alleles. These mutations, however, differ in the way that they interact with putative selector genes, previously shown to influence both the signal and the response components of the eyespot developmental mechanism in homologous eyespots positioned either on the fore or on the hindwings. *Spotty *targets eyespots only on the forewing and, therefore, may be under Ubx gene regulation, whereas *Missing *has a Ubx-gene independent effect, and reduces the size of eyespots on both fore and hindwing. Both these mutation are acting quite early in the eyespot development pathway by affecting the differentiation of the central group of cells, the focus, responsible for early pupal signaling and differentiation of the concentric rings of colored scales in an eyespot [29,30].

These two mutations highlight the ability of genes of large effect to affect subsets of serially homologous structures and give them a separate identity from the rest. It is still unclear, however, whether the ancestral condition for these serial homologues is that of separately controlled units, regulated by genes such as *Spotty *and *Missing *that have very discrete affects on eyespot development brought together to form a complete row of eyespots, or whether, on the other hand, eyespots appeared as a cohesive unit of similarly looking structures, one per wing cell, resembling the proposed Nymphalid Groundplan [13], that gradually gained separate regulatory control by the appearance of genes such as *Spotty *and *Missing*. Phylogenetic comparative methods may eventually be able to shed light on this question.

## Methods

### Crosses

We reared a series of single pair families by placing virgin adult butterflies in small cubic net hanging cages and followed the crossing scheme outlined in Table 5. In addition, we used data from previously reared single pair crosses of wildtype with wildtype [12], reared in the same climate room and in identical conditions to the new crosses described here. Adults were reared on banana, eggs were collected on young maize plants, and larvae from each family were reared in tube-like net cages on maize plants. All animals were reared inside a climate room at 27°C, 12 h:12 h light:dark photoperiod and 80% relative humidity. Upon emergence the ventral wing pattern of F1 individuals was photographed with a black and white video camera and images were saved as Tiff files.

**Table 5:** Summary of all the crosses performed between Spotty (S), Missing (M), and Wildtype (Wt) individuals.

**Crosses**	**Family**	**# of offspring**
**Spotty (male) crosses:**		
S male (PmABC2) × Wt female (PfA2)	A2	53
S male (PmABC3) × Wt female (PfA3)	A3	33
S male (PmABC7) × Wt female (PfB7)	B7	20
S-Wt hybrid male (PmB72) × Wt female (Pf72)	B72	68
S-Wt hybrid male (PmB73) × M female (PfB73)	B73	45
		
**Wild type (male) crosses:**		
Wt male (PmDE2) × S female (PfD2)	D2	44
Wt male (PmDE3) × S female (PfD3)	D3	34
		
Wt male (PmDE3) × M female (PfE3)	E3	25
		
Wt male × Wt female	16*	84
Wt male × Wt female	17*	35
Wt male × Wt female	19*	76
Wt male × Wt female	20*	62
Wt male × Wt female	51*	64
		
**Missing (male) crosses**		
M male (Pm1) × M female (Pf1)	1	10
M male (Pm4) × M female (Pf4)	4	5
M male (Pm6) × M female (Pf6)	6	41
M male (Pm7) × M female (Pf7)	7	13
M male (Pm11) × M female (Pf11)	11	3
M male (Pm14) × M female (Pf14)	14	15
M male (Pm15) × M female (Pf15)	15	24
M male (Pm16) × M female (Pf16)	16	13
M male (Pm18) × M female (Pf18)	18	17
M male (Pm20) × M female (Pf20)	20	12
M male (Pm21) × M female (Pf21)	21	6
M male (Pm22) × M female (Pf22)	22	30
		
M male (PmFGH2) × Wt female (PfF2)	F2	73
M male (PmFGH3) × Wt female (PfF3)	F3	28
M male (PmFGH4) × Wt female (PfF4)	F4	49
M male (PmFGH5) × Wt female (PfF5)	F5	46
M-Wt hybrid male (PmF51) × M female (PfF51)	F51	70
M-Wt hybrid male (PmF52) × M female (PfF52)	F52	58
M-Wt hybrid male (PmF55) × M female (PfF55)	F55	27
		
M male (PmFGH2) × S female (PfG2)	G2	25
M male (PmFGH3) × S female (PfG3)	G3	8
M male (PmFGH5) × S female (PfG5)	G5	44
M-S hybrid male (PmG27) × Wt female (PfG27)	G27	52
M-S hybrid male (PmG28) × Wt female (PfG28)	G28	60
M-S hybrid male (PmG51) × Wt female (PfG51)	G51	129
M-S hybrid male (PmG56) × Wt female (PfG56)	G56	84
M-S hybrid male (PmG59) × Wt female (PfG59)	G59	99
M-S hybrid male (PmG511) × Wt female (PfG511)	G511	119
M-S hybrid male (PmG112) × Wt female (PfG512)	G512	48
		
M-S hybrid male (PmG55) × S female (PfG55)	G55	54
		
M-S hybrid male (PmG33) × M female (PfG33)	G33	82
M-S hybrid male (PmG35) × M female (PfG35)	G35	27
M-S hybrid male (PmG36) × M female (PfG36)	G36	60
M-S hybrid male (PmG53) × M female (PfG53)	G53	91
M-S hybrid male (PmG54) × M female (PfG54)	G54	83
M-S hybrid male (PmG57) × M female (PfG57)	G57	141
M-S hybrid male (PmG58) × M female (PfG58)	G58	141
M-S hybrid male (PmG513) × M female (PfG513)	G513	43
M-S hybrid male (PmG514) × M female (PfG514)	G514	73
M-S hybrid male (PmG515) × M female (PfG515)	G151	89

(* data from Monteiro et al. 2003).

The ventral pattern of forewings and hindwings was quantified by measuring the diameter of the black discs of all eyespots present on the ventral side of fore and hindwings along an axis parallel to the wing veins. Diameter measurements were done using the Tiff files in Object Image 1.62 [31]. Data were later transferred to MS Excel version X.

### Statistics

Analyses of variance for eyespot size were performed by asking whether there was a significant size difference for any of the forewing or hindwing eyespots (analyzed individually) between offspring from different types of cross (Missing × Wildtype, Wildtype × Wildtype, etc). Cross type was used as a fixed factor. For all analyses sex was also used as a fixed factor, since males are usually smaller than females and also have smaller eyespots, and family was coded as a random factor, nested within cross type. Analyses were done using SPSS version 11 for the Macintosh. Regression analyses were done in Excel version X for the Macintosh. Graphics were done is SPSS and in Excel.

### Immunohistochemistry

Late fifth instar larval wings of Missing individuals were stained according to the protocol described in [32] with antibodies targeting Distal-less (a gift from Grace Boekhoff-Falk [33]) and Engrailed (antibody 4F11, a gift from Nipam Patel [34]).

### Association study

An additional cross was performed between a Wildtype male and a Missing female, followed by a backcross of a single F1 male offspring to another Missing female. The bodies of these individuals were separated from the wings, placed in 2 ml screw cap vials filled with 100% ethanol, and stored at -70°C. Hindwings for all the backcross progeny were photographed, and the diameter of eyespots at positions 3 and 4 was measured and averaged for each individual. DNA was extracted from the four parents, and a 690 bp 5'UTR fragment, from the previously cloned *Distal-less *(*Dll*) gene [35] was amplified with the following two primers: Dll-NF1 (5'-CGCGAGTTGGTTGTGTCGGGTTACCTCGGA-3') and Dll-R6 (5'-CGTGGAAACACAGCATCACTATCACA-3'). PCR amplification conditions were 5 min at 94°, followed by 35 cycles of 40 sec at 94°C, 40 sec at 57°C, and 60 sec at 72°C. The amplification ended with a 5 min final extension period at 72°C. The PCR product was inserted into pGEM-T vector (Promega) and cloned in *E. coli *JM109 cells. Five clones were sequenced from each of the four parents and the sequences were screened for the presence of an informative polymorphic marker, i.e, a marker with two alleles and with a different allele, ideally in homozygote condition, present in the Missing and Wildtype grandparents. This marker should also be easily genotyped by means of an enzymatic essay where homozygote and heterozygote individuals can be differentiated by their restriction-digest banding pattern. The backcross generation was genotyped by first extracting DNA from the most extreme 15 individuals from each of the two ends of the eyespot size distribution. The same *Dll *fragment was amplified via PCR from these individuals, purified using a Quiagen PCR purification column, and then digested overnight with BseNI (BsrI) restriction enzyme following the manufacture's directions (Fermentas). Digested DNA was run on a 1.5% agarose gel, photographed, and the genotype was assayed from the restriction pattern.

## Authors' contributions

AM designed and coordinated the experiments, helped rear the butterflies, collected the immunohistochemistry data, analyzed the eyespot size data, and wrote the manuscript. BC, assisted by LV, performed the *Dll *association study, LS scanned all the wings, performed all the eyespot measurements, and assisted AM in the eyespot size analyses, JP did most of the butterfly rearing, AB prepared the butterfly wings for imaging and the bodies for DNA analysis, and PMB helped design the experiments and critically revised the final manuscript.
